# Intrathoracic extramedullary hematopoiesis presenting as tumor-simulating lesions of the mediastinum in α-thalassemia: A case report

**DOI:** 10.3892/ol.2015.3597

**Published:** 2015-08-12

**Authors:** JUN AN, YIMIN WENG, JINYUAN HE, YUN LI, SHAOHONG HUANG, SONGWANG CAI, JUNHANG ZHANG

**Affiliations:** Department of Cardiothoracic Surgery, The Third Affiliated Hospital, Sun Yat-Sen University, Guangzhou, Guangdong 510630, P.R. China

**Keywords:** mediastinal tumor, extramedullary hematopoiesis, thalassemia

## Abstract

Extramedullary hematopoiesis (EMH) is a rare disease, where hematological disorder drives extramedullary hematopoietic tumor formation in multiple regions of the body. The present study reports a case of EMH presenting as multiple tumor-like lesions of mediastinum in a 61-year-old male with α-thalassemia, which was subjected to a video-assisted thoracoscopic surgery tissue biopsy to differentiate it from other mediastinal tumors. To date, only three cases of EMH in patients with α-thalassemia have been described in the literature. Patients with EMH typically exhibit no hematological disorder preoperatively and therefore EMH is frequently misdiagnosed. In the present study, along with a literature review of the clinicopathological features of EMH, the diagnosis and treatment of this rare case was discussed, in order to differentiate diagnosis, and particularly to distinguish EHM from extramedullary myeloid sarcoma.

## Introduction

Extramedullary hematopoiesis (EMH), which refers to hematopoiesis occurring outside the medulla of the bone, is a rare hematological disease, secondary to insufficient bone marrow function (1,2). EMH occurs under conditions of myelofibrosis and ineffective erythropoiesis, including that in thalassemia, hereditary spherocytosis and sickle cell disease (3–5). While EMH may occur anywhere in the body, it most commonly presents as diffuse lesions in the liver, spleen and/or lymph nodes. In rare cases, EMH presents as a solitary mass (6), appearing as a tumor-simulating lesion in an atypical location, and is frequently misdiagnosed.

Alpha-thalassaemia is inherited as an autosomal recessive disorder characterized by a microcytic hypochromic anaemia, and is a result of impaired production of 1,2,3, or 4 alpha globin chains, leading to a relative excess of beta globin chains of haemoglobin (Hb) (7). It is one of the most common monogenic gene disorders worldwide and is particularly frequently observed in Mediterranean countries, South-East Asia and Africa. As a compensatory response in various anaemias, EMH may develop with thalassaemia (7).

The present study describes a case of EMH presenting as multiple tumor-like lesions in the mediastinum of a patient with α-thalassemia. The patient was diagnosed with mediastinal EMH following a tissue biopsy via video-assisted thoracoscopic surgery (VATS). The study protocol was approved by the Research Ethics Committee of The Third Affiliated Hospital, Sun Yat-Sen University (Guangzhou, China).

The present case report discusses the major diagnostic problems of mediastinal EMH, distinct characteristics for differentiating EMH from other mediastinal tumors and the treatment strategy applied, as well as a literature review of this rare clinical entity.

## Case report

**Figure 1.**
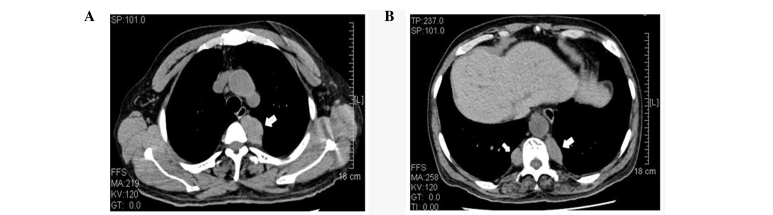
Extramedullary hematopoiesis appears as a solid homogeneous mass (green arrow) in the posterior mediastinal on a thoracic computed tomography scan. (A) T3-4 vertebral level. (B) T10 vertebral level.

A 61-year-old Chinese male presented with a 3-year history of intermittent chest pain and tightness. Physical examination revealed elevated blood pressure (149/96 mm Hg), but no other remarkable findings. A chest X-ray and computed tomography (CT) scan were performed and multiple paravertebral masses were identified (Fig. 1A). Subsequently, a contrast-enhanced CT scan of the chest revealed multiple bilateral mediastinal masses, observed as round or oval, smooth, soft-tissue opacities immediately lateral to the thoracic vertebra. The mass in the left thoracic paravertebral space at the T3-T4 vertebral level measured 44×25 mm, and the two masses on the right of the T10 vertebra measured 52×48×17 mm and 32×22 mm (Fig. 1B). In addition, the initial CT scan results indicated the possibility of neurogenic tumors.

The complete blood count revealed the following results: White blood cells, 11.44×10^9^/l; hemoglobin (Hb), 119 g/l; platelets, 176×10^9^/l; mean corpuscular volume, 76.4 fl; mean corpuscular Hb concentration, 258 g/l; segmental neutrophils, 48.1%; lymphocytes, 44.5%; monocytes, 5% and eosinophils, 1.9%. No pathological changes were detected in erythrocyte sedimentation rate or C-reactive protein.

**Figure 2.**
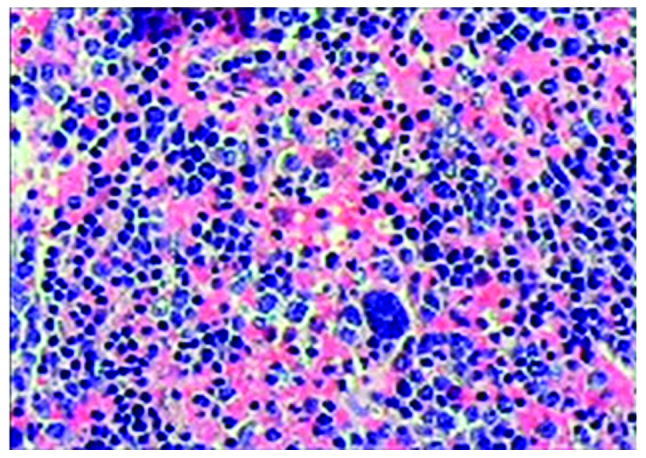
EMH microscopic findings. A focus of EMH, characterized by the presence of hematopoietic cells, erythrocytes and megakaryocytes, in the resected specimen (hematoxylin and eosin stain; magnification, x100). EMH, extramedullary hematopoiesis.

To determine the diagnosis of the mediastinal masses, a surgical tissue biopsy via VATS was performed following the provision of consent of the patient and family. A section of the dark-red mass in the left thoracic cavity was excised and electrocoagulation was used for hemostasis. Frozen section examination revealed EMH. A conventional permanent histopathological examination was also performed; the biopsied tissue was stained (hematoxylin and eosin) and examined under a BX43 microscope (Olympus Corporation, Tokyo, Japan), revealing hyperplasia with activity of two types of hematopoietic cell, namely erythroid and megakaryocytic cells (Fig. 2). Furthermore, immunohistochemical staining indicated that diffuse cells from the mediastinal lesions were positive for CD43, CD99 and high Ki-67, while sporadic cells were positive for myeloperoxidase, CD15, CD3, CD45RO and CD61. The diffuse and sporadic cells were negative for creatine kinase, CD30 and Pax-5. Myeloid sarcoma, a rare extramedullary form of myelogenous leukemia, also known as granulocytic sarcoma or chloroma was therefore excluded. Based on these pathological and clinical findings, the patient was diagnosed with thoracic EMH.

Postoperatively, various laboratory tests were performed, which demonstrated low Hb (90 g/l). Subsequently, thalassemia-associated tests were performed. A diagnosis of α-thalassemia was made based on the increased glucose-6-phosphate dehydrogenase activity (15919.000 mU/g), decreased HbA2 (0.9%) and RBC-BT (erythrocyte fragility test; 29.0%), as well as positive hemoglobin H staining with hemoglobin electrophoretic studies (HbH, 10.7%).

**Figure 3.**
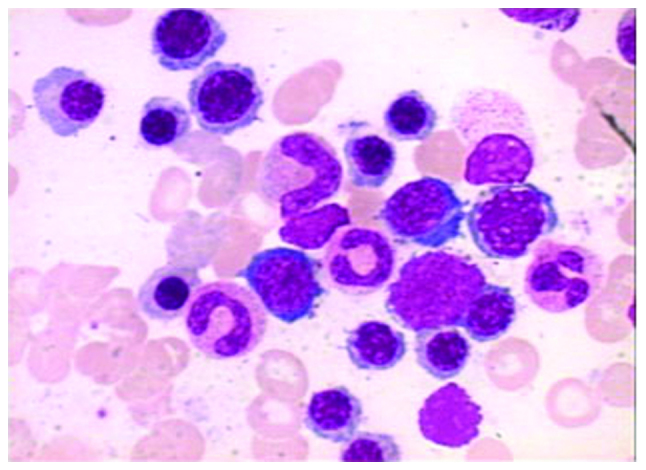
Bone marrow aspirate analysis. A bone marrow aspirate revealed erythroid hyperplasia (G:E, 0.82:1), and the relative proportion of immature red blood cells was also increased but no malignant cell invasion was observed. (Wright-Giemsa stain; magnification, x1,000).

The patient was transferred to the Department of Hematology for further treatment, where bone marrow aspiration cytology confirmed the findings of anemia and erythroid hyperplasia (G:E, 0.82:1). In addition, the relative proportion of immature red blood cells was also significantly increased, but was not accompanied by malignant cell invasion (Fig. 3).

**Figure 4.**
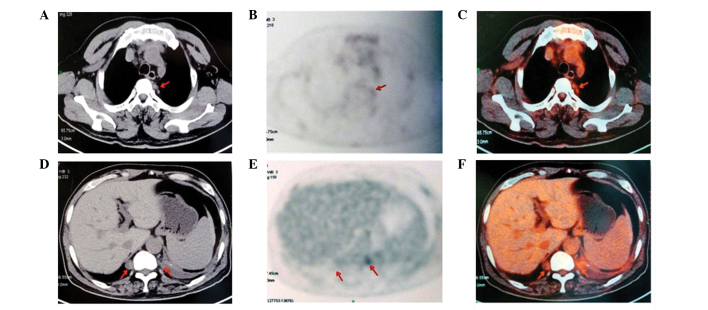
EMH at the posterior-superior mediastinum (T3-T4 vertebral level) and the posterior-inferior mediastinum (T10 vertebral level) on a postoperative PET/CT scan (red arrow). (A) CT image, (B) PET image and (C) fused image at T3-T4 vertebral level. PET/CT indicated slightly increased FDG uptake in mediastinal mass at the T3-T4 vertebral level. (SUV_max_, 5.2; SUV average, 2.8). (D) CT image, (E) PET image and (F) fused PET/CT image at the T10 vertebral level detected EMH as a benign-appearing mass. SUV_max_ was markedly decreased in EMH (SUV_max_, 3.8; SUV average, 2.4). EMH, extramedullary hematopoiesis; PET/CT, positron emission tomography-computed tomography; FDG, 18-fluorodeoxyglucose.

Subsequently, 18-fluorodeoxyglucose (FDG) positron emission tomography-computed tomography (PET/CT) was performed to evaluate the mediastinal masses and determine the presence of any additional extramedullary masses. PET/CT identified remnant tissue in the left thoracic T3-T4 paravertebral space following biopsy, and the maximum standardized uptake value (SUV_max_) was 5.2. The remaining two paravertebral masses, at the posterior-inferior mediastinum at the T10 level, demonstrated no increased glucose metabolism, with SUV_max_ values of 3.8 (Fig. 4).

Since the postoperative course following VATS was uneventful and the patient exhibited no clinical symptoms, regular outpatient follow-up observation was selected, rather than treatment. At 8 months postoperatively, at the most recent follow-up visit, the patient exhibited no evidence of disease.

## Discussion

For a primary mediastinal mass, the potential clinical differential diagnoses are extensive. However, EMH, a rare entity involving the formation and development of blood cells outside of the bone marrow, is an unlikely diagnostic candidate.

EMH occurs in response to the failure of erythropoiesis in bone marrow and may occur in myeloproliferative disorders, hemoglobinopathies or bone marrow infiltration. EMH most commonly affects the spleen and liver and, occasionally, the lymph nodes. Less commonly involved organs include the pleura, lungs, gastrointestinal tract, breast, skin, brain, kidneys and adrenal glands (8). The occurrence of EMH as a tumor-simulating mass in the intrathoracic cavity, particularly a posterior mediastinal mass, is very rare (9). A review of the literature revealed that only three cases of EMH in patients with α-thalassemia have been reported, to date (5,10,11).

EMH is frequently characterized by the development of soft tissue masses in the paravertebral thoracic regions. These masses rarely induce significant symptoms, but may result in hemothorax and pleural effusion (5,12). In the present case, the patient was asymptomatic and EMH was identified by chance.

Patients with no hematologic disorder, presenting with such masses induced by EMH, are frequently misdiagnosed (9,13). EMH is often incidentally diagnosed, when patients undergo evaluation for unrelated symptoms, as in the present case. While EMH diagnosis requires a biopsy, a needle biopsy carries the risk of hemothorax. In the presence of anemia, a diagnosis of EMH may be considered preoperatively; however, in the present case, the primary blood tests identified Hb as 119 g/l; therefore, EMH was initially overlooked as a potential diagnosis.

Notably, extramedullary myeloid sarcoma (EMS) is also a rare extra medullary tumor mass consisting of myeloid blasts. EMS is also known as granulocytic sarcoma or chloroma, since it is diagnosed based on the presence of a green color caused by myeloperoxidase. EMS occurs in ~5% of patients with acute myeloid leukemia (AML) (14). Rarely, EMS may predate AML, presenting as an isolated mass in various sites of the body. However, EMS in the mediastinal region has rarely been reported (15,16). EMS is primarily diagnosed based on immunohistochemical staining of tissue biopsy. Although EMH and EMS arise from distinct hematological diseases, the two conditions should be considered as differential diagnoses for a mediastinal tumor.

A PET/CT scan may be diagnostically useful in such cases. To date, only a few cases regarding the diagnosis of EMH using PET/CT have been reported (17,18), with EMH detected as a benign mass with low SUV_max_ values and normal appearance of the tissue. The concurrent presence of an underlying hematopoietic disorder may suggest a diagnosis of EMH. In EMS, a PET/CT scan identifies intense FDG uptake (16).

As a result of its rarity, there are no evidence-based guidelines for the treatment of thoracic pseudotumors induced by EMH. Essentially, EMH is a compensatory mechanism for chronic anemia; therefore, recurrent blood transfusions to correct such anemia may result in shrinkage of the mass. However, blood transfusions are not entirely harmless or risk-free, and this therapeutic effect is temporary, slow to occur and may be insufficient (19–21). Hematopoietic tissue is notably radiosensitive and undergoes shrinkage with radiotherapy; thus, excellent results have been obtained in cases of EMH with a few days of radiotherapy alone (22,23). Surgical excision is required in patients with EMH associated with complications, including bleeding from the mass (9), spinal cord compression or spinal canal invasion (13). Such excision does not only staunch the bleeding and/or allow decompression but also provides a histological diagnosis. Following surgical excision, radiotherapy may aid the prevention of recurrence of EMH or facilitate complete remission (24,25).

Based on the literature review, it was hypothesized that close follow-up of the patient was important. Considering the excellent results obtained with low-dose radiotherapy for EMH masses, the patient will be advised to undergo radiation therapy in the future.

EMH should be considered in the differential diagnosis of an intrathoracic mass. Particularly, if a history of thalassemia or chronic anemia is discovered, based on evaluation of the patient's medical history, as well as laboratory studies. Surgical resection and radiation therapy should be considered as treatment options for EMH.
